# HYDROPS FETALIS ASSOCIATED WITH HOMOZYGOSITY FOR Hb ADANA [α59(E8)Gly→Asp (α2)]

**DOI:** 10.3109/03630269.2010.493405

**Published:** 2010-08-19

**Authors:** Ita M. Nainggolan, Alida Harahap, Iswari Setianingsih

**Affiliations:** The Eijkman Institute for Molecular Biology, Jakarta, Indonesia

**Keywords:** Hydrops fetalis, α-Thalassemia (α-thal), Hb Adana [α59(E8)Gly→Asp (α2)], Molecular diagnosis

## Abstract

We describe cases of hydrops fetalis associated with nondeletional α-thalassemia (α-thal), in three unrelated Indonesian families. The genotypes of the fetuses and their parents were generated by DNA sequencing and by a polymerase chain reaction restriction fragment length polymorphism (PCR-RFLP)-based method to rapidly identify mutations detected by sequencing. Two of the fetuses had hydrops fetalis and homozygous α59(E8)Gly→Asp (α2), also known as Hb Adana. The third fetus was also suspected to be homozygous for Hb Adana because both parents were carriers of this mutation. This study shows that homozygosity for Hb Adana is associated with hydrops fetalis in the Indonesian population. We discuss this mutation and its various phenotypes including compound heterozygosity with other α-thal mutations and describe a simple approach to genetic testing that will clarify the risk of hydrops fetalis in the offspring of couples carrying this nondeletional mutation.

In Southeast Asia, homozygosity for Southeast Asian-type α^0^-thalassemia (α^0^-thal), (– –^SEA^/– –^SEA^) is the most common cause of hydrops fetalis (1-3). This paper examines three cases of hydrops fetalis in Indonesian fetuses homozygous for the HBA2:c.179G>A mutation (also known as Hb Adana or the α^codon 59^α/α^codon 59^α genotype); one mother was a compound heterozygote for the Hb Adana mutation and a “silent” mutation at codon 22 of the α2 gene [α22(B3)Gly→Gly or HBA2:c.69C>T; also known as the α^codon 59^α/α^codon 22^α genotype] and she manisfested a moderate α-thal. The codon 59 mutation is also known as Hb Adana and was first described on the α1-globin gene [α59(E8)Gly→Asp (α1)] in two Turkish patients (4). The same mutation was subsequently found on the α2-globin gene [α59(E8)Gly→Asp (α2)] in an Albanian patient (5).

Two sets of parents carrying hydrops fetalis fetuses in a current pregnancy and one set with a previous history of hydrops fetalis were referred to the GenNeka Clinic (Yayasan GenNeka, Eijkman Institute for Molecular Biology, Jakarta, Indonesia) for genetic evaluation and counseling. All are ethnically Javanese.

Two cases of hydrops fetalis were diagnosed by ultrasonography. Complete blood counts (Cell Dyne 1700; Abbot Diagnostics, Abbott Park, IL, USA), erythrocyte morphology examination by microscope and hemoglobin (Hb) analysis by high performance liquid chromatography (HPLC) on a VARIANT™ Hemoglobin Testing System (β-Thalassemia Short Program; Bio-Rad Laboratories, Hercules, CA, USA) (6) were performed for all fathers, mothers and available newborn babies as well as affected fetuses. We did not assess the iron status of all individuals. II-1 (Family 1) and II-1 (Family 2) were delivered at the gestational ages of 22 weeks and 21 weeks, respectively. Cordocentesis was performed on both II-1 (Family 1) and II-1 (Family 2), who died after delivery; prenatal diagnosis was performed on II-2 (Family 1) and II-1, II-2 and II-3 (Family 2). The pedigrees are shown in Figure 1. To eliminate maternal cell contamination in the prenatal diagnosis, DNA from the parents and the fetuses was fingerprinted for variable number tandem repeat (VNTR) D1S80 and apolipoprotein B (APO-B), (7,8).

**FIGURE 1:**
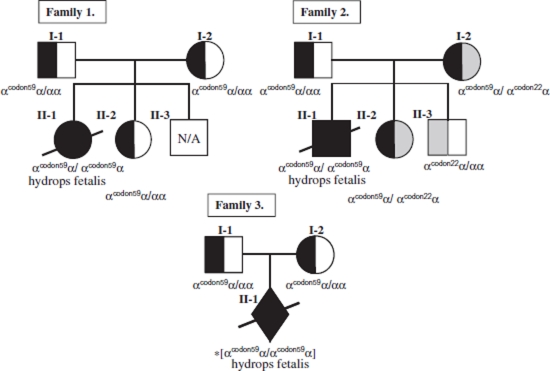
Pedigrees and genotypes of the three family members. *Presumed genotype based on parental genotypes; N/A = not available.

DNA was extracted from peripheral blood leukocytes from all six parents and from the cord blood of II-1 and II-2 (Family 1), II-1, II-2 and II-3 (Family 2). DNA was isolated using a modification of the Puregene DNA isolation method (Gentra Systems Inc., Minneapolis, MN, USA). DNA from amniocytes was isolated using a salting-out method adapted from Miller et al. (9). For all parents, DNA analysis consisted of multiplex polymerase chain reaction (m-PCR) for α^0^-thal deletions including the Southeast Asian, Filipino, and Thai types (10). Multiplex PCR for α^0^-thal types was negative for all family members. Direct DNA sequencing of the whole α2- and α1-globin genes was performed using the BigDye Dideoxy Terminator sequencing kit v3.1 (Applied Biosystems, Foster City, CA, USA) on an ABI PRISM™ 3130 automated sequencing (Applied Biosystems). DNA sequencing carried out on the parents in Family 1 and the mother in Family 2 (I-2), showed that they were all heterozygous for Hb Adana on the α2-globin gene. Then we developed a direct mutation detection system using a PCRRFLP (restriction fragment length polymorphism) method. Since there is no restriction site that can differentiate between the normal and mutant alleles, we developed an ACRS (amplified created restriction site) method to directly detect the codon 59 (α2) mutation. The forward primer, codon 59modF, was a modified primer where *T* (thymine) was used to introduce a recognition sequence for the Taq-^α^I restriction enzyme in the mutant allele. The forward and reverse primers, codon 59modF (5′-GCT CTG CCC AGG TTA AGG GCC *T*CG-3′) and α2R (5′-GGG AGG CCC ATC GGG CAG GAG GAA C-3′) (11), were used to amplify a 460 bp fragment (GenBank, NG_000006). The PCR reaction mixture (25 μL) contained 100 ng DNA, 10 μM of each primer, 200 μM dNTPs, 1X PCR buffer and 1.25 units of Taq DNA polymerase (New England Biolabs, Beverly, MA, USA). Reactions were carried out in a PE 9700 thermocycler (Perkin Elmer Applied Biosystems, Foster City, CA, USA) with an initial denaturation of 5 min. at 95°C followed by 35 cycles at 95°C for 30 seconds, 68°C for 30 seconds and 72°C for 1 min and one cycle at 72°C for 5 mins. The PCR product was digested using Taq-^α^I restriction enzyme (New England Biolabs) and visualized under UV light (Gel Doc; Bio-Rad Laboratories). Figure 2 shows an example of gel electrophoresis of the PCR-RFLP products. The PCR-RFLP analysis for this mutation showed all parents were heterozygous for Hb Adana (α2) and DNA sequencing on both α2- and α1-globin genes confirmed that the PCR-RFLP method correctly identified the genotypes (data not shown). In addition, DNA sequencing of I-2 (Family 2) showed that she also carried a synonymous α22(B3)Gly→Gly (α2) in trans allele as well as the codon 59 (α2) mutation. The α22(B3)Gly→Gly (α2) is predicted to produce an abnormal splice donor site between codons 22 and 23 (GGTGAG) leading to a premature termination between codons 48 and 49 (12). I-2 (Family 2), who is a compound heterozygote for both these mutations, shows a more severe phenotype compared to those with only Hb Adana (α2). This result provides evidence that the α22(B3)Gly→Gly (α2) is indeed a thalassemia allele.

**FIGURE 2:**
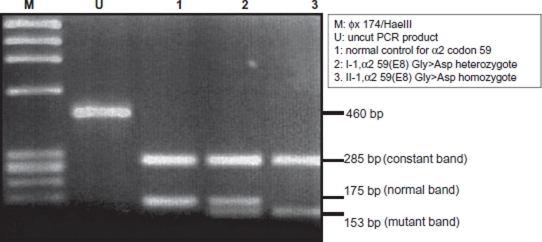
Gel electrophoresis of the PCR-RFLP products from Family 1.

The II-1 (Family 1) and II-1 (Family 2) fetuses were homozygous for the Hb Adana (α2) mutation. II-1 (Family 3) was delivered dead at 25 weeks gestation and samples were not obtained. Given the parental genotypes, the death of the fetus was assumed to be due to homozygosity for Hb Adana (α2). The II-1 (Family 1) and II-1 (Family 2) fetuses, who were homozygous for Hb Adana (α2), produced peaks on the HPL chromatogram that corresponded with Hb Bart's (γ_4_), Hb H (β_4_), Hb A (α2β2) and Hb F (α2γ2) (13,14). Hematological data for all cases are shown in Table 1.

**TABLE 1:** Hematology Profiles and Genotypes of the Three Family Members

Subjects	Sex-age	Hb (g/dL)	MCV (fL)	MCH (pg)	RDW (%)	Hb A_2_ (%)	Hb F (%)	Hb Types	Genotypes
Family 1									
I-1	M-32	15.2	75.3	25.8	15.1	2.8	0.5	A, A_2_, F	α^codon 59^α/αα
I-2^a^	F-28	10.7	63.5	21.3	22.6	2.7	1.5 A, A_2_, F	α^codon 59^α/αα	
II-1	F-(22)^b^	2.9	82.0	37.2	40.2	0.2	93.2	Bart's, H, A, F	α^codon 59^α/α^codon 59^α
II-2	F-1	day	ND	ND	ND	ND	ND	ND	ND
Family 2									
I-1	M-30	15.9	75.8	25.2	15.9	2.6	0.3	A, A_2_, F α^codon 59^α/αα	
I-2	F-24	9.4	73.2	24.5	16.7	1.9	3.0	A, A_2_, F	α^codon 59^α/α^codon 22^α
I-2^c^	F-25	7.6	73.5	23.1	20.2	ND	ND	ND	α^codon 59^α/α^codon 22^α
II-1	M-(21)^b^	1.3	ND	ND	ND	0.0	83.6	Bart's, H, A, F	α^codon 59^α/α^codon 59^α
II-2	F-1 day	ND	ND	ND	ND	ND	67.5	ND	α^codon 59^α/α^codon 22^α
II-3	M-1 day	16.0	89.2	30.8	16.2	0.0	84.9	A, A2, F	α^codon 22^α/αα
Family 3									
I-1	M-28	13.7	75.3	24.1	17.2	3.0	0.0	A, A2, F	α^codon 59^α/αα
I-2^d^	F-26	13.6	71.4	23.4	16.4	2.4	0.0	A, A2, F	α^codon 59^α/αα
II-3	?-(25)^b^	ND	ND	ND	ND	ND	ND	ND	ND
Normal Values									
Adults	>18	12.0–18.0	80.0–97.0	27.0–31.0	12.0–15.0	2.3–3.5	<1.0	A, A_2_, F	
Newborns		15.0–18.0	88.0–114.0	33.0	15.0–19.0	0.0	>90.0	A, F	

Hb: hemoglobin; MCV: mean corpuscular volume; MCH: mean corpuscular Hb; RDW: red blood cell distribution width; ND: Not done.

a These values are for I-2 (Family 1) 3 weeks after delivering the hydrops fetalis fetus.

b These fetuses were miscarried at 22, 21 and 25 (gender unknown) weeks gestation.

c These values are for I-2 (Family 2) at 15 weeks gestation.

d These values are for I-2 (Family 3) 1 month after having delivered a hydrops fetalis fetus and receiving a blood transfusion.

I-2 (Family 1) and I-2 (Family 2), with different genotypes, both showed Hb levels lower than normal during pregnancy, however, I-2 (Family 1) never required blood transfusions, whereas I-2 (Family 2) always required blood transfusion 4 to 5 times during each pregnancy. We do not know the cause of the mild anemia of I-2 (Family 1) because we did not assess the iron level. The other mother, I-2 (Family 3), had blood drawn for hematological examination 1 month after she had received a blood transfusion for postpartum hemorrhage after delivering a hydrops fetalis fetus and showed a normal Hb level of 13.6 g/dL. The clinical manifestation of individuals who are heterozygous for Hb Adana (α2) during pregnancy in these cases is still unclear. The clinical manifestations of the Hb Adana carriers in general are within normal limits, with normal Hb levels and slightly reduced MCV and MCH levels as shown in Table 1.

The cases in this study are of interest because fetuses with the α^codon 59^α/ α^codon 59^α genotype all manifest as hydrops fetalis although they still have two functional α-globin genes. Moreover, the clinical manifestations of these hydrops fetalis fetuses were more severe (miscarried at 22, 21 and 25 weeks gestation) than those of fetuses with the – –^SEA^/– –^SEA^ or α^codon 59^α/–– genotype, who died at about 33 weeks (range 23–43 weeks) (15) and at 28–29 weeks gestational age, respectively (16,17). Interestingly, the compound heterozygote for Hb Adana on the α1-globin gene and α^0^-thal did not manifest as hydrops fetalis but severe hemolytic anemia which required regular blood transfusion from an early age (4,18). This might be caused by the higher rate of the α2-globin gene transcription than the α1-globin gene that resulted in the higher amount of variant α-globin chain or unstable variant Hb. Although we still do not know the mechanism of hydrops fetalis in a fetus with the codon 59 (α2) homozygosity, because they still have two intact α-globin genes on chromosome 16, it appears that the severity of the phenotype might not be due to the decreased α-globin chains synthesis but the variant α-globin chains interfere with normal tetramer formation or damage erythrocytes in some other way. It has also been suggested that this unstable Hb variant has a defect in the detoxification process by α-Hb stabilizing protein (19). Therefore, the higher amount of variant α-globin chain or unstable variant Hb will result in a more severe phenotype. Further research is required to elucidate how this mutation causes hydrops fetalis. Hydrops fetalis due to other non deletional α-thalassemias (homozygosity for α-globin variants has been previously reported) (20,21), however it was unclear whether it was the α-thal alone which caused hydrops fetalis in both cases.

While reports of Hb Adana (α2 or α1) from other countries are limited, in our Clinic, the recent frequency of this mutation seems high (16%) in Indonesian α-thal patients with clinically manifested thalassemia intermedia and thalassemia major including hydrops fetalis cases (unpublished data). Our previous population study (14), based on hematology parameters found the carrier frequency of α^0^- and α^+^-thal in the Indonesian population to be around 2.6–3.2% and 2.7–11%, respectively. DNA analysis limited to large α-globin gene deletions (two and one α-globin gene), showed that the frequency of the 3.7 kb deletion was around 0.5–2%, and that of the 4.2 kb deletion about 0.2%. We found that the frequency of the Hb Adana (α2) mutation is quite high (14). The possibility of a high frequency of the Hb Adana (α2) mutation in Indonesians and the severe phenotype of this mutation in the homozygous state is a very important issue in the prevention and management program of thalassemia in Indonesia. Moreover, most Hb Adana carriers are asymptomatic or exhibit only mild anemia, with red cell indices quite similar to those of α^+^-thal carriers due to one α-globin gene deletion (14). Therefore, molecular diagnosis should be proposed as a routine diagnosis for all suspected α-thal carriers. For this purpose, the PCR-RFLP method that has been developed can be used as a less expensive and faster method.
